# Supplementation of glutamine in a short-term boar semen extender during 17°C holding time enhances post-thaw sperm quality for cryopreservation

**DOI:** 10.1186/s40813-024-00403-8

**Published:** 2024-11-11

**Authors:** Morakot Nuntapaitoon, Padet Tummaruk, Junpen Suwimonteerabutr

**Affiliations:** 1 Department of Obstetrics, Gynaecology and Reproduction, Faculty of Veterinary Science, Chulalongkorn University, Bangkok, 10330 Thailand; 2 Center of Excellence in Swine Reproduction, Chulalongkorn University, Bangkok, 10330 Thailand

**Keywords:** Boar sperm, Cryopreservation, Glutamine, Holding time, Post-thaw

## Abstract

**Background:**

Glutamine is a nonessential amino acid and the most abundant amino acid found in the seminal plasma and sperm-rich fraction of boar semen. Glutamine plays an important role in enhancing glutathione (GSH) synthesis. It acts as an effective antioxidant in semen and provides intracellular defense to sperm against oxidative stress. This study aimed to improve the quality of frozen-thawed boar semen by using glutamine supplementation in a short-term semen extender during the holding time at 17 °C before cryopreservation.

**Results:**

The results indicate that the total motility, progressive motility, LIN, STR, and WOB were the highest in the 20 mM supplementation group at the 2 h timepoint after thawing. Thus, the optimal concentration for glutamine supplementation in short-term boar semen extender during the holding time at 17 °C was 20 mM. Interestingly, at all of the time points after thawing, 20 mM glutamine supplementation exhibited the highest level of sperm viability and membrane integrity when compared to the CONTROL (0 mM) and other experimental dilution groups. Moreover, the acrosome integrity, mitochondrial activity, and capacitation status (F pattern) were significantly greater in the 20 mM supplementation group than the other groups at the 2 h timepoint after thawing.

**Conclusion:**

Supplementation of glutamine at a concentration of 20 mM in a short-term semen extender (Bio Pig^®^) during the 17 °C holding time before cryopreservation, which had a standard freezing extender (9.0% glycerol and 1.9% Equex paste), could enhance the post-thaw sperm motility and quality parameters of cryopreservation.

## Background

The process of artificial insemination in pig industries is widespread, and the use of chilled semen provides a higher pregnancy rate when compared with cryopreserved semen [1, 2]. However, semen cryopreservation must be performed to facilitate the movement and preservation of a wide range of commercially important genetic traits. The harmful effects of semen cryopreservation can be reduced by the improvement of semen extenders and cryopreservation techniques. Thus, various cryopreserved semen extenders have been proposed to protect sperm from damaging factors, such as freeze and osmotic shock, oxidative stress, and cell injury by ice crystals [3].

Cryoprotective agents can be divided into permeating and nonpermeating agents. Permeating agents are frequently used, including DMSO, glycerol, 1–2 propanediol, and ethylene glycol, which help enhance amorphous glass formation tendencies to decrease the likelihood of ice nucleation and intracellular ice formation [4]. Nonpermeating agents do not pass through a sperm plasma membrane and act at the extracellular level [5]. These include egg yolk, sugars, amino acids, various proteins, lipoproteins, methyl cellulose, polyvinyl alcohol, polyvinylpyrrolidone, hydroxyethyl starch, and dextran, which supports the prevention of ice formation and stabilizes the proteins and cellular membranes [6, 7].

Glutamine is a nonessential amino acid and the most abundant amino acid found in the seminal plasma and sperm-rich fraction of boar semen [8]. Glutamine plays an important role in enhancing glutathione (GSH) synthesis. It acts as an effective antioxidant in semen and provides intracellular defense to sperm against oxidative stress. Oxidative stress has a detrimental effect on sperm function due to increased lipid peroxidation induced by reactive oxygen species (ROS) during the process of freezing and thawing [9–11]. Antioxidant supplementation is highly beneficial because the post-thaw boar sperm quality is believed to be due to an imbalance in ROS generation, and the antioxidant-mediated reduction ameliorates this [1, 12].

Fresh semen from boar ejaculate is rich in antioxidants that are essential to creating a natural redox balance for the generation and reduction of ROS [13, 14]. During the process of cryopreservation, the boar ejaculates are centrifuged to eliminate the seminal plasma-derived antioxidants or reduce the ejaculates to a smaller volume [2]. Thus, the natural defense from ROS is removed and often not substituted when a freezing extender is added. The total or partial removal of the seminal plasma results in sperm that have less motility and metabolic activity and reduced fertility capacity [15]. Thus, the addition of seminal plasma or components of seminal plasma, especially proteins and antioxidants, may enhance sperm cryotolerance [16].

Previous studies have investigated the addition of glutamine at a concentration of 80 mM and 2% glycerol to a freezing extender for boar semen cryopreservation to determine whether it could improve post-thaw sperm motion parameters compared to a control (3% glycerol). However, a standard freezing extender (20% egg yolk and 3% glycerol) supplemented with glutamine at concentrations of 20–80 mM did not exhibit significant improvement in the post-thaw sperm parameters [17]. Nonetheless, Wang et al. [18] found that Beltsville Thawing solution (BTS) supplemented with glutamine provided a greater protective effect on boar sperm against oxidative stress by enhancing GSH synthesis during liquid storage at 17 °C. To date, no studies have investigated glutamine supplementation in short-term extenders during holding time exposure before the freezing process. The purpose of this study is to improve the sperm quality of freeze-thawed boar semen by using glutamine supplementation in a short-term boar semen extender during the holding time at 17 °C before cryopreservation.

## Materials and methods

### Animals

Twenty-one ejaculates from 11 mature boars (six Duroc, two Landrace, and three Yorkshire) were collected at a boar stud in Ratchaburi province and Livestock Animal Hospital, Faculty of Veterinary Science, Chulalongkorn University, Nakhon Pathum province. All boars were proven sires and aged between 1 and 3 years. The boars were kept in individual pens (9 m^2^ per boar) in a closed housing enclosure equipped with an evaporative cooling system. Each boar was fed 2.5–3.2 kg of commercial feed daily as a standard diet, and water was provided ad libitum via water nipples. This study was reviewed and approved by the Institutional Animal Care and Use Committee (approval number 1931086), Faculty of Veterinary Science, Chulalongkorn University, Bangkok, Thailand.

### Experimental design

For the 21 ejaculates from the 11 boars included in the experiment, each ejaculate was diluted in a semen extender, Bio Pig^®^ (Megapor, Zaragoza, Spain), to which four different concentrations of glutamine (Sigma, Brazil) (0 (CONTROL), 20, 40, 80, and 100 mm) were added at 37 °C, cooled, and held for 3 h at 17 °C. The freezing process was then performed as detailed below. After being stored in liquid nitrogen (− 196 °C) for 1 month, the 0.5-mL straw was thawed at 50 °C for 12 s, followed by a post-thaw incubation period (0 h (10 min), 1 h, and 2 h). Sperm quality analysis including sperm motility, viability, acrosome integrity, mitochondrial activity, membrane integrity, and capacitation status were evaluated.

### Semen collection and freezing process

Boar semen was collected using a glove-handed method. Sperm concentration (range: 100–600 × 10^6^ sperm/mL) was measured using a Spermacue^®^ (Minitübe, Tiefenbach, Germany). Ejaculates with ≥ 70% subjective motility and ≥ 90% normal morphology were included in the experiment. The semen was cryopreserved according to our previously described protocol. Briefly, the semen was diluted (1:1–2 v/v) in Bio Pig^®^ (Megapor) extender (Extender I), to which four different concentrations of L-glutamine (G3126, Sigma) (0 (CONTROL), 20, 40, 80, and 100 mm) were added at 37 °C within 10 min of collection and transported to the laboratory in a cool insulated foam box maintained at an ambient temperature of 15–17 °C [19]. The diluted semen was cooled and stored in a controlled-temperature refrigerator (LIEBHERR, Bulle, Switzerland) at 17 °C for a further hold time of 3 h. Thereafter, the semen was centrifuged at 800 ×*g* for 10 min at 17 °C. The sperm pellet was diluted in Tris-egg yolk (TEY) extender (111 mM Tris, 31.4 mM citric acid, 185 mM glucose, and 20 mL of egg yolk) (Extender II) [17] to a concentration of 1.5 × 10^9^ sperm/mL. The semen was then cooled to 5 °C for 2 h. Extender III (TEY, 9.0% glycerol, and 1.9% Equex paste (Minitübe, Tiefenbach, Germany)) was added to the extended semen to a final concentration of 1 × 10^9^ sperm/mL. Finally, the extended semen was loaded into 0.5-mL PVC-French straws (IMV Technologies, France). In the freezing process, the straws were placed 4 cm above the liquid nitrogen vapor for 20 min and were then plunged into the liquid nitrogen (− 196 °C). The straws were kept in a nitrogen tank for at least 1 month before thawing [20, 21].

### Thawing process

The straws were thawed in 50 °C water for 12 s [22] and dissolved in Bio Pig^®^ (Megapor) extender to a concentration of 2 × 10^9^ sperm/mL [23]. Analysis of the post-thaw sperm quality was determined after incubation at 37 °C for 0 h (10 min), 1 h, and 2 h.

## Sperm quality analysis

### Sperm motility

#### Computer-assisted sperm analysis (CASA)

Total sperm motility, progressive motility, and sperm kinematics including straight-line velocity (VSL, µm/s), curvilinear velocity (VCL, µm/s), and average path velocity (VAP, µm/s), linearity (LIN (%); the ratio between VSL and VCL), straightness (STR (%); the ratio between VSL and VAP), wobble (WOB, oscillation index; the ratio between VAP and VCL), amplitude lateral head (ALH, µm), and beat frequency (BCF, Hz) were evaluated using a computer-assisted sperm analysis system (SCA^®^ CASA System, MICROPTIC S.L., Barcelona, Spain). The module for boar sperm in the CASA system was set and applied as follows: frame rate (fps), 50; static (µm/s), < 10; slow-medium (µm/s), 25; rapid (µm/s), > 45; progressive (STR), 45; connectivity (pixels), 11; and VAP points (pixels), 5. The diluted semen was placed into the chamber and examined on a warmed stage (TOKAI HIT, Shizuoka-ken, Japan) at 37 °C under a phase-contrast microscope (CX-41, Olympus, Tokyo, Japan). The proportion of motile sperm was quantified from 1,000 sperm cells in five randomly selected fields for each sample [18, 24].

## Sperm viability

Sperm viability was evaluated using SYBR-14 with a LIVE/DEAD™ Sperm Viability Kit (L7011; Thermo Fisher Scientific, USA). The post-thawed semen samples were diluted with phosphate-buffered saline (PBS) in a 1:10 ratio. A 10 µL aliquot of the diluted semen sample was combined with 10 µL of 4.65 µM EthD-1 (Ethidium Homodimer-1) (Thermo Fisher Scientific, USA) and 2.7 µL of the SYBR-14 working solution (1:100 v/v in DMSO) and then incubated in a block heater at 37°C for 15 min. One drop (8 µL) of the stained sperm sample was placed onto a glass slide and covered with a coverslip. Two hundred spermatozoa were examined using a fluorescence microscope (BX-50; Olympus) (400×). Spermatozoa that only stained green fluorescence were classified as live sperm. Spermatozoa that stained red or green and red fluorescence were regarded to be dead sperm, respectively [25].

### Acrosome integrity

Acrosome integrity was measured using EthD-1 (Ethidium Homodimer-1) (Thermo Fisher Scientific, USA) and fluorescein isothiocyanate-labeled peanut (*Arachis hypogaea*) agglutinin (FITC-PNA) staining (Sigma-Aldrich Co. Ltd., St Louis, MO, USA). The post-thawed semen samples were diluted with PBS in a 1:10 ratio. A 10 µL diluted semen sample was combined with 10 µL of 4.65 µM EthD-1 and incubated in a block heater at 37 °C for 15 min. After incubation, one drop (10 µL) of combined semen sample was smeared on a slide and air-dried at 20–25 °C. The dried slide was fixed with 95% ethanol for 30 s before staining with 50 µL of 100 µg/mL FITC-PNA solution (1 mg/mL FITC-PNA in PBS (1:10, v/v)) at 4 °C for 30 min in a moist chamber, then washed with cold PBS (4 °C), and air-dried at 20–25 °C. Two hundred spermatozoa per sample were evaluated by fluorescence microscopy (BX-50; Olympus) (1,000×). The proportion of sperm with an intact acrosome, i.e., with a green-stained acrosome cap (positive), was calculated. Sperm cells were without an acrosome cap, with a green band at the equatorial segment, or with a disrupted patch-like appearance of the acrosome cap, were considered negative [25].

### Mitochondrial activity

Mitochondrial activity was assessed using fluorochrome 5,5′,6,6′-tetrachloro-1,1′,3,3′-tetraethylbenzimidazoly-carbocyanine iodide (JC-1; (Thermo Fisher Scientific, USA). Briefly, the post- thawed semen samples were diluted with PBS in a 1:10 ratio. The diluted semen sample (12.5 µL) was combined with 25 µL of JC-1 solution (1.6 µL of 0.153 mM JC-1, 1 µL of 0.02 mM SYBR-14, and 1.6 µL of 2.4 mM propidium iodide in 100 µL HEPES-buffered medium) and incubation in a block heater at 37 °C for 30 min. Then, one drop (8 µL) of stained sperm sample was placed onto a glass slide and covered with a coverslip. Two hundred spermatozoa were evaluated using a fluorescent microscope (BX-50; Olympus) (400×). Spermatozoa with yellow-orange fluorescence at the midpiece were classified as positive with high mitochondrial membrane potential, whereas spermatozoa with green or less green fluorescence at the midpiece were regarded as negative with low mitochondrial membrane potential [25, 26].

### Membrane integrity

Membrane integrity was determined using the short hypo-osmotic swelling test (HOS test). A 10 µL semen sample was combined with 200 µL of citrate buffer (75 mOsM) and incubated in a block heater at 37 °C for 30 min. After incubation, 50 µL of HOS solution with 5% formaldehyde (75 mOsM) was added. One drop (8 µL) of sperm sample was placed onto a glass slide and covered with a coverslip. The appearance of the tails of 200 spermatozoa was evaluated using phase-contrast microscopy (CX-31; Olympus) (400×) and classified as positive (sperm with a coiled tail) or negative (sperm with a straight tail). The percentage of positive sperm is indicative of the functional sperm membranes [25].

### Capacitation status

**Fig. 1:**
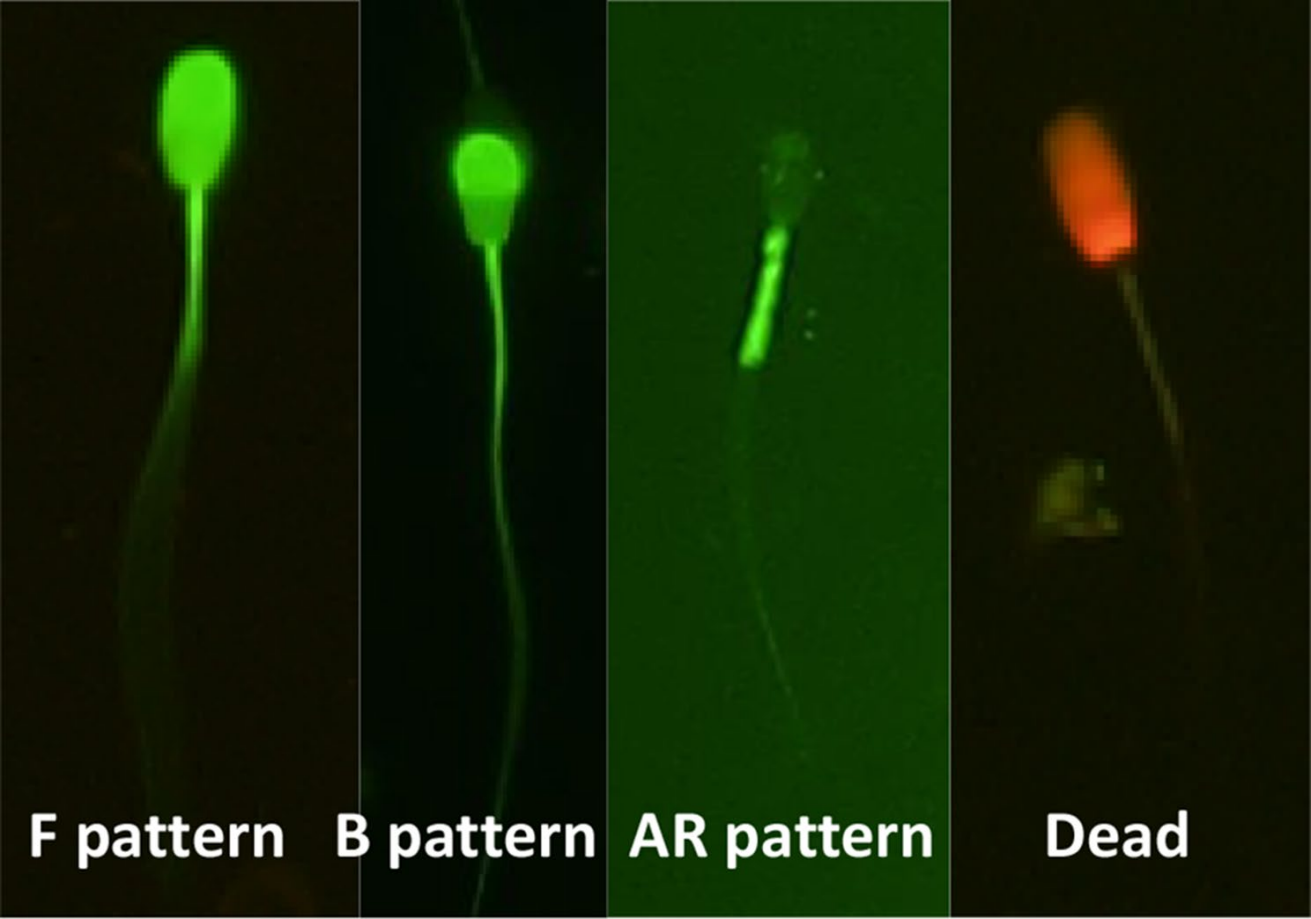
Four patterns obtained from the CTC/6-CFDA/EthD-1 staining of boar spermatozoa. F = live/noncapacitated spermatozoa; B = live/capacitated spermatozoa; AR = acrosome-reacted spermatozoa; and Dead = red color of the whole sperm head (EthD-1 positive)

Capacitation status was assessed using chlortetracycline CTC (750 µM CTC (C4881; Sigma-Aldrich), 130 mM NaCl, 5 mM L-cysteine, and 20 mM Tris-HCl [pH 7.8]) according to the protocol previously reported by Grasa et al. [27], with some modifications. First, the post- thawed semen samples were diluted with PBS in a 1:10 ratio. Thereafter, 48 µL of the diluted sperm sample was combined with 2 µL of 23.34 µM Ethidium homodimer-1 (Molecular Probes Europe) and 0.5 µL of 6-carboxyfluorescein diacetate (6-CFDA) (C5041; Sigma-Aldrich) in concentration at 0.46 mg/mL, which was then incubated in a block heater at 37°C for 10 min. After incubation, the mixture was stained with 20 µL of CTC solution and fixed with 1 µL of 4% paraformaldehyde. Finally, one drop (6 µL) was placed onto a glass slide and combined with 2 µL of 0.22 M antifade triethylenediamine (DABCO; Sigma-Aldrich). The sample was then covered with a coverslip and pressed under tissue paper to create a thin layer and absorb any excess fluid. The prepared slide was kept in the dark until evaluation. Two hundred spermatozoa were examined using a fluorescent microscope (BX-50; Olympus) (400×). There were four sperm types: F pattern (whole sperm head shows bright fluorescence, with/without a brighter equatorial band; this is indicative of intact or non-capacitated spermatozoa); B pattern (the acrosomal region of the sperm head fluoresces brightly but the post-acrosomal region does not; this indicates a capacitated acrosome-intact spermatozoa); AR pattern (the acrosomal region of the sperm head is nonfluorescent, with/without a fluorescent post-acrosomal region; this indicates a capacitated acrosome-react spermatozoa); and Dead (the whole sperm head is a red color; this indicates dead spermatozoa (EthD-1 positive)), which are shown in Fig. 1. Two hundred spermatozoa per slide were counted. Only the percentage of the three patterns obtained from the CTC-stained viable spermatozoa (EthD-1 negative) was determined in this experiment.

### Statistical analysis

Statistical analyses were performed using SAS (SAS version 9.4, Cary, NC, USA). All sperm motility characteristics were analyzed by multiple ANOVA using the SAS general linear mixed model procedure (MIXED). The statistical models included a fixed effect of group (0 (CONTROL), 20, 40, 80, and 100 mm glutamine), time (0 h (10 min), 1 h, and 2 h), and their interaction. Boar was included in the models as a random effect. The least squares means were obtained from the statistical models and were compared using the least significant difference test. *P* < 0.05 was considered statistically significant.

## Results

Between the group and time in the statistical model for sperm motility and kinematic parameters after thawing and incubation at 37 °C for 0 h (10 min), 1 h, and 2 h, there were significant decreases for the individual parameters (*P* < 0.001) during storage, but it did not affect the group × time with regards to LIN, STR, WOB, and ALH (*P* > 0.05) (Table 1).

**Table 1:** Significant levels of the individual parameters after thawing and incubation at 37^o^C with the group and time

Parameter	Group	Time	Group × Time
Total motility	< 0.001	< 0.001	< 0.001
Progressive motility	< 0.001	< 0.001	< 0.001
Rapid velocity	< 0.001	< 0.001	< 0.001
VCL	< 0.001	< 0.001	0.016
VSL	< 0.001	< 0.001	0.002
VAP	< 0.001	< 0.001	0.002
LIN	< 0.001	< 0.001	0.358
STR	< 0.001	< 0.001	0.703
WOB	< 0.001	< 0.001	0.241
ALH	< 0.001	< 0.001	0.170
BCF	< 0.001	< 0.001	0.007

### Sperm quality evaluation of frozen-thawed sperm

**Fig. 2:**
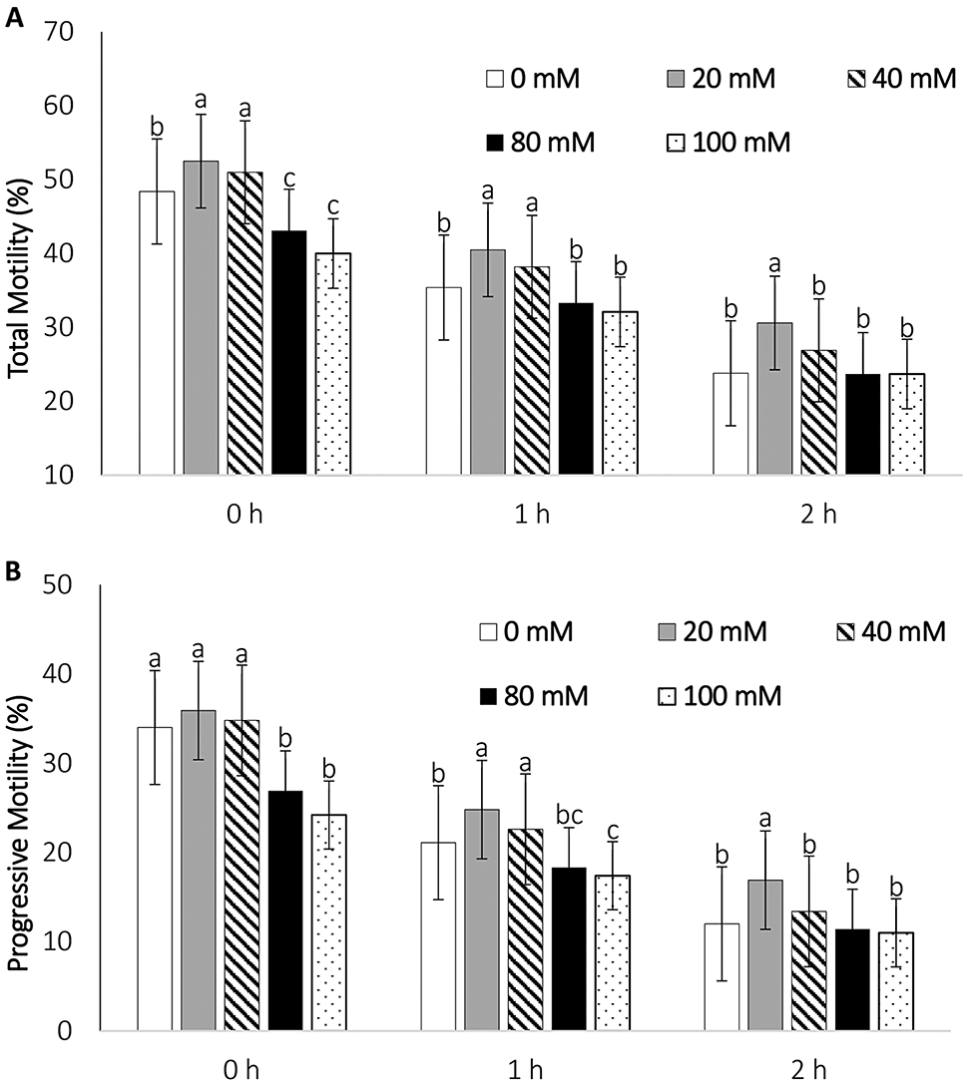
Effect of glutamine supplementation at different concentrations (0 (CONTROL), 20, 40, 80, and 100 mM) on the total motility (**A**) and progressive motility (**B**) in each group at 0 h (10 min post-thawing), 1 h, and 2 h

**Fig. 3:**
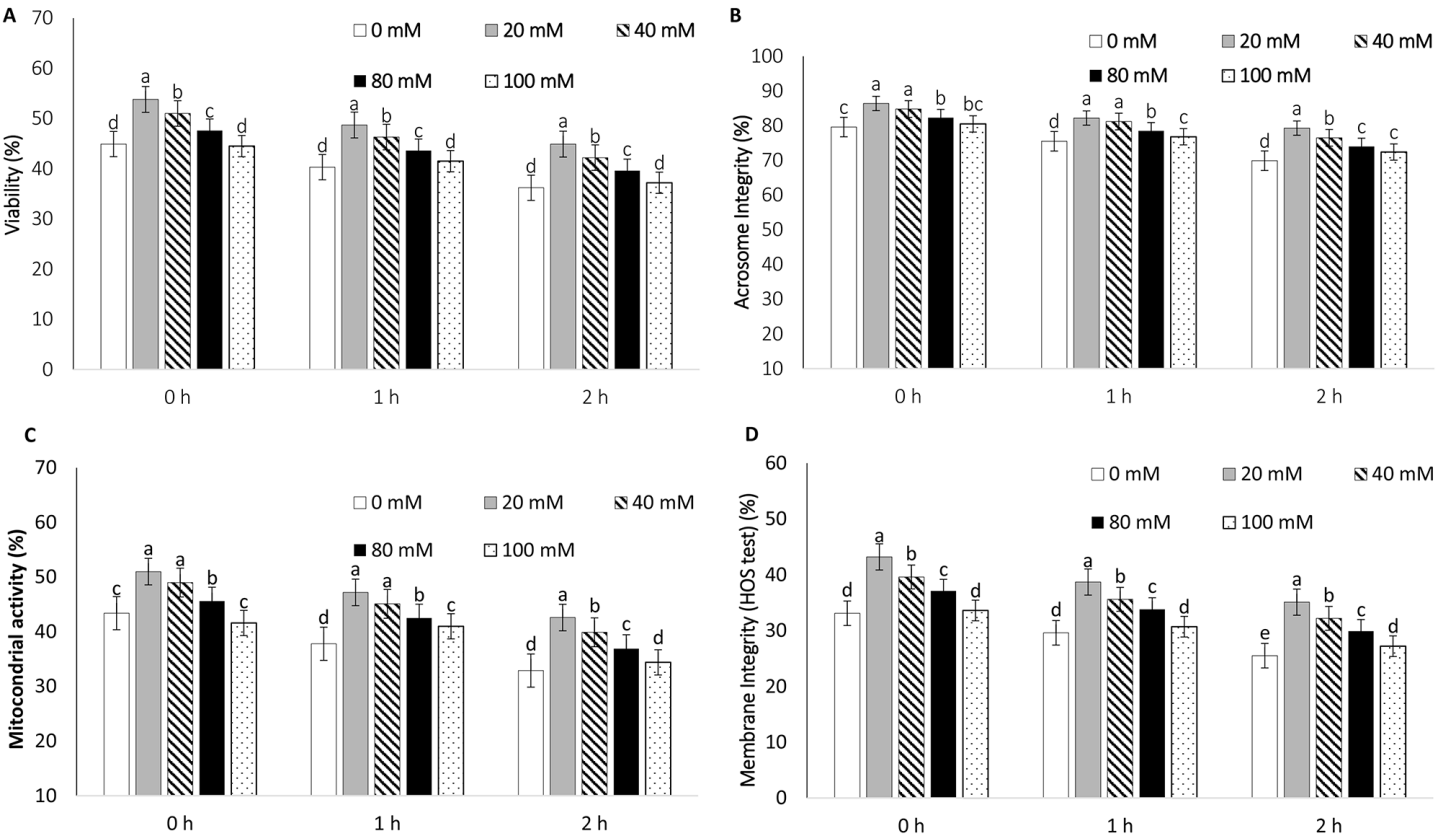
Effect of glutamine supplementation at different concentrations (0 (CONTROL), 20, 40, 80, and 100 mM) on sperm viability (**A**), acrosome integrity (**B**), mitochondrial activity (**C**), and membrane integrity (HOS test) (**D**) at 0 h (10 min post-thawing), 1 h, and 2 h. ^a, b, c, d^ Significant difference within the groups (*P* < 0.05)

The total motility and progressive motility were higher in the short-term semen extender (Bio Pig^®^) supplemented with 20 mM glutamine during the holding time at 17 °C than in the CONTROL (Fig. 2). Moreover, LIN, STR, and WOB were higher in the 20 mM glutamine supplementation group than in the other groups after thawing and incubation at 37 °C for 2 h (Table 2). In all the experimental groups, the kinematic parameters decreased over time after being stored at 37 °C (Table 2). Glutamine supplementation at 20 mM increased the tested sperm quality parameters compared with the CONTROL (Fig. 3). The capacitation status in the F pattern of the post-thawed semen in the 20 mM glutamine supplementation group was highest among the various time periods at 37 °C (Table 3).

**Table 2:** Least squares mean (LSM) ± standard error of the mean (SEM) of the sperm kinematics in frozen-thawed semen stored for various time periods (0 h (10 min), 1 h, and 2 h) at 37 °C analyzed by CASA

Time	Group	Parameter								
		Rapid	VCL	VSL	VAP	LIN	STR	WOB	ALH	BCF
0 h	0 mM	27.5 ± 1.3^a^	63.5 ± 1.4^a^	23.7 ± 1.2^a^	36.9 ± 1.3^a^	32.3 ± 1.7^ab^	52.7 ± 1.5^ab^	53.7 ± 1.7^ab^	1.64 ± 0.03^a^	7.9 ± 0.4^a^
	20 mM	28.5 ± 1.3^a^	60.9 ± 1.4^a^	23.3 ± 1.2^a^	35.9 ± 1.3^a^	33.0 ± 1.7^a^	53.6 ± 1.5^a^	54.7 ± 1.7^a^	1.61 ± 0.03^ab^	7.4 ± 0.4^a^
	40 mM	28.1 ± 1.3^a^	61.3 ± 1.4^a^	22.7 ± 1.2^a^	35.3 ± 1.3^a^	31.6 ± 1.7^a^	52.2 ± 1.5^ab^	52.9 ± 1.7^ab^	1.61 ± 0.03^ab^	7.4 ± 0.4^a^
	80 mM	19.7 ± 1.3^b^	54.9 ± 1.4^b^	18.5 ± 1.2^b^	30.0 ± 1.3^b^	29.5 ± 1.7^b^	50.4 ± 1.5^b^	50.7 ± 1.7^b^	1.54 ± 0.03^b^	6.1 ± 0.4^b^
	100 mM	18.6 ± 1.3^b^	54.5 ± 1.4^b^	16.3 ± 1.2^b^	28.1 ± 1.3^b^	25.5 ± 1.7^c^	46.6 ± 1.5^c^	46.9 ± 1.7^c^	1.32 ± 0.03^b^	5.4 ± 0.4^b^
1 h	0 mM	15.7 ± 1.3^ab^	49.9 ± 1.4^a^	14.2 ± 1.2^abc^	24.0 ± 1.3^ab^	24.8 ± 1.7^b^	47.0 ± 1.5^ab^	44.2 ± 1.7^b^	1.44 ± 0.03	5.4 ± 0.4^a^
	20 mM	18.0 ± 1.3^a^	49.8 ± 1.4^a^	15.8 ± 1.2^a^	25.6 ± 1.3^a^	28.2 ± 1.7^a^	49.4 ± 1.5^a^	48.0 ± 1.7^a^	1.43 ± 0.03	5.6 ± 0.4^a^
	40 mM	16.6 ± 1.3^a^	49.9 ± 1.4^a^	14.6 ± 1.2^ab^	24.4 ± 1.3^ab^	25.5 ± 1.7^b^	46.9 ± 1.5^ab^	44.9 ± 1.7^ab^	1.43 ± 0.03	5.4 ± 0.4^ab^
	80 mM	13.4 ± 1.3^b^	47.6 ± 1.4^ab^	13.4 ± 1.2^bc^	23.0 ± 1.3^bc^	24.7 ± 1.7^b^	45.6 ± 1.5^b^	44.6 ± 1.7^b^	1.41 ± 0.03	4.7 ± 0.4^bc^
	100 mM	12.5 ± 1.3^b^	46.7 ± 1.4^b^	12.2 ± 1.2^c^	21.6 ± 1.3^c^	22.8 ± 1.7^b^	44.4 ± 1.5^b^	42.8 ± 1.7^b^	1.40 ± 0.03	4.5 ± 0.4^c^
2 h	0 mM	8.3 ± 1.3^b^	44.5 ± 1.4^ab^	10.2 ± 1.2^ab^	19.0 ± 1.3^ab^	19.7 ± 1.7^b^	42.3 ± 1.5^b^	38.6 ± 1.7^b^	1.34 ± 0.03^a^	3.8 ± 0.4^ab^
	20 mM	11.6 ± 1.3^a^	45.9 ± 1.4^a^	12.1 ± 1.2^a^	21.5 ± 1.3^a^	24.4 ± 1.7^a^	45.7 ± 1.5^a^	44.3 ± 1.7^a^	1.39 ± 0.03^a^	4.2 ± 0.4^a^
	40 mM	9.2 ± 1.3^ab^	44.0 ± 1.4^ab^	9.7 ± 1.2^b^	18.5 ± 1.3^b^	19.7 ± 1.7^b^	41.9 ± 1.5^b^	38.6 ± 1.7^b^	1.35 ± 0.03^a^	3.7 ± 0.4^ab^
	80 mM	7.6 ± 1.3^b^	42.1 ± 1.4^b^	9.1 ± 1.2^b^	17.2 ± 1.3^b^	19.5 ± 1.7^b^	41.4 ± 1.5^b^	38.5 ± 1.7^b^	1.22 ± 0.03^b^	3.4 ± 0.4^b^
	100mM	7.7 ± 1.3^b^	42.8 ± 1.4^b^	8.9 ± 1.2^b^	17.5 ± 1.3^b^	18.0 ± 1.7^b^	39.4 ± 1.5^b^	36.7 ± 1.7^b^	1.32 ± 0.03^a^	3.4 ± 0.4^b^

^a, b^ Different superscript letters within rows indicate significant differences (*P* < 0.05)

**Table 3:** Least squares mean (LSM) ± standard error of the mean (SEM) of the capacitation status of the post-thawed semen from 21 ejaculates in each group at various time periods (0 h (10 min post thawing), 1 h, and 2 h) with different concentrations of glutamine supplementation (0 (CONTROL), 20, 40, 80, and 100 mM) at 37 °C

Time	Group	Pattern			
		F	B	AR	
0 h	0 mM	22.6 ± 1.2^d^	9.8 ± 0.9	11.3 ± 1.0	
	20 mM	33.9 ± 1.2^a^	9.9 ± 0.9	11.3 ± 1.0	
	40 mM	30.4 ± 1.2^b^	9.5 ± 0.9	10.8 ± 1.0	
	80 mM	27.3 ± 1.2^c^	9.6 ± 0.9	9.7 ± 1.0	
	100 mM	23.5 ± 1.2^d^	9.0 ± 0.9	10.4 ± 1.0	
1 h	0 mM	16.7 ± 1.2^c^	7.3 ± 0.9^b^	12.3 ± 1.0	
	20 mM	26.3 ± 1.2^a^	8.6 ± 0.9^ab^	11.8 ± 1.0	
	40 mM	24.5 ± 1.2^a^	8.4 ± 0.9^ab^	10.7 ± 1.0	
	80 mM	20.8 ± 1.2^b^	9.4 ± 0.9^a^	11.0 ± 1.0	
	100 mM	18.5 ± 1.2^bc^	9.0 ± 0.9^ab^	11.0 ± 1.0	
2 h	0 mM	12.4 ± 1.2^d^	6.3 ± 0.9^b^	11.8 ± 1.0	
	20 mM	21.4 ± 1.2^a^	8.7 ± 0.9^ab^	12.7 ± 1.0	
	40 mM	18.8 ± 1.2^b^	8.2 ± 0.9^ab^	11.0 ± 1.0	
	80 mM	17.0 ± 1.2^bc^	9.1 ± 0.9^a^	9.3 ± 1.0	
	100 mM	15.2 ± 1.2^c^	7.5 ± 0.9^ab^	10.0 ± 1.0	

^a, b, c^ Different superscript letters within a column indicate significant differences (*P* < 0.05)

### Effects of different concentrations of glutamine supplementation in a short-term extender during the holding time and time after thawing on boar sperm motility

Total and progressive motility was the highest in a short-term semen extender (Bio Pig^®^) supplemented with 20 mM glutamine during the holding time at 17 °C before the freezing process at 2 h after thawing (*P* < 0.5) (Fig. 2). Immediately after thawing (0 h (10 min)), the total motility in the 20 mM glutamine group (52.5%) was greater than that in the CONTROL (48.4%, *P* = 0.013), 80 mM (43.1%, *P* < 0.001), and 100 mM (41.1%, P *<* 0.001) groups, but it was not significantly different from the 40 mM group (51%, P *>* 0.05) (Fig. 2). Moreover, after thawing and incubation at 37 °C for 2 h, the total motility in the 20 mM supplementation group (30.6%) was significantly greater than that in the CONTROL (23.9%, P *<* 0.001), 40 mM (26.9%, *P* = 0.023), 80 mM (23.7%, P *<* 0.001), and 100 mM (23.7%, P *<* 0.001) supplementation groups.

The CASA results of the post-thawed (0 (CONTROL), 20, 40, 80, 100 mm) glutamine supplementation groups in a short-term boar semen extender and stored at 37 °C for 0 h (10 min), 1 h, and 2 h revealed that the progressive motility in the 20 mM group (35.9%) was greater than that in 80 mM (26.9%, *P <* 0.001) and 100 mM (24.2%, P *=* 0.042) groups, but this difference was not significant when compared with the CONTROL group (0 mM) (*P* > 0.05). At 2 h after thawing, progressive motility in the group with 20 mM glutamine supplementation was significantly greater than the other groups (P *<* 0.05) (Fig. 2).

### Effects of different concentrations of glutamine supplementation in a short-term extender during the holding time and the time after thawing on boar sperm kinematics

At 2 h post-thawing, LIN, STR, and WOB were the highest in the 20 mM glutamine supplementation group among the other groups (P *<* 0.05). Moreover, the 20 mM group had a higher rapid velocity than the CONTROL group (0 mM) at 2 h post thawing (Table 2). However, the 20 mM glutamine supplementation group did not affect sperm kinematics immediately when compared with the CONTROL and 40 mM groups (P *>* 0.05), but it was higher than the 80 mM and 100 mM supplementation groups (P *<* 0.05). At 1 h after thawing, the LIN and WOB parameters in the 20 mM glutamine group were higher compared with the CONTROL group (P *<* 0.05) (Table 2).

### Effects of different concentrations of glutamine supplementation in a short-term extender during the holding time and the time after thawing on boar sperm quality

The effects of a short-term semen extender supplemented with 20 mM glutamine during the holding time before freezing on sperm quality are presented in Fig. 3. Interestingly, at all of the measured time points after thawing, 20 mM glutamine supplementation exhibited the highest sperm viability (P *<* 0.05) and membrane integrity (HOS test) (P *<* 0.05) among the other groups. (Fig. 3). Acrosome integrity and mitochondrial activity in the 20 mM glutamine group at 0 h (10 min) and 1 h were greater than the CONTROL and other experimental groups except the 40 mM group. However, acrosome integrity and mitochondrial activity in the 20 mM supplementation group were the highest among all of the other groups after thawing at 2 h (P *<* 0.05) (Fig. 3).

Furthermore, capacitation status in the F pattern of the 20 mM glutamine supplementation group (33.9%) at 0 h (10 min) was greater than those in the CONTROL (22.6%), 40 mM (30.4%), 80 mM (27.3%), and 100 mM (23.5%) groups (Table 3). After thawing for 2 h, the capacitation status in the F pattern of the 20 mM glutamine supplementation group (21.4%) was the greatest among those in the CONTROL (12.4%), 40 mM (18.8%), 80 mM (17%), and 100 mM (15.2%) (*P* < 0.05) groups (Table 3).

## Discussion

Glutamine has an alpha-amino acid structure and is a component of glutathione, which has a major role in protecting sperm cells against ROS [10]. Ahmad et al. [28] demonstrated that glutamine has a cryoprotective effect during the freeze-thaw process of goat sperm. Moreover, glutamine enhanced the protective effect of glycerol during the freeze-thaw process by having a synergistic effect with glycerol on stallion semen cryopreservation [29].

Holding time is defined as the storage period at 15°C–17°C between sperm collection and the start of the cryopreservation process with further cooling to 5°C. The holding time increases sperm cryotolerance by maintaining the lipid architecture of the plasma membrane [30]. This facilitates greater sperm viability and fertility [31]. This experiment shows that glutamine supplementation has a cryoprotective effect during the freeze-thaw process of boar sperm, as 20 mM glutamine supplementation in a short-term boar semen extender (Bio Pig^®^) increased the post- thaw sperm motility when compared to the CONTROL and other experimental groups after thawing for 2 h. Moreover, the results from this study indicate that the sperm trajectory as determined by CASA, especially LIN, STR, and WOB, in the 20 mM glutamine supplementation group after thawing for 2 h, was higher than the other groups (*P* < 0.5). Previously, several reports regarding LIN have revealed that it is a significant indicator in swine reproduction trials. Boar semen that had sperm movement exhibiting increased VSL and LIN was correlated with larger little sizes after insemination [5, 32]. Tremoen et al. [33] reported that LIN on the day of collection and WOB after storage influenced the total number of piglets born in Norwegian Landrace in a swine artificial insemination study.

Herein, 20 mM glutamine supplementation in the experimental groups during the holding time at 17°C before the freezing process tended to have higher sperm quality with regards to sperm viability, acrosome integrity, mitochondria activity, membrane integrity, and capacitation status than the CONTROL group except for the 100 mM glutamine group. The optimal concentration of glutamine in this study was 20 mM, and the 20 mM supplementation group had the best performance in all time points for sperm viability and membrane integrity (*P* < 0.5). Moreover, in the 20 mM supplementation group, the acrosome integrity and mitochondrial activity were greater than the other groups after thawing for 2 h (*P* < 0.5). Similarly, Zhu et al. [11] indicated that 20 mM glutamine supplemented in a freezing extender significantly improved sperm motility, acrosome integrity, membrane integrity, and mitochondrial activity. Several studies have reported that the optimal concentration of glutamine was 5 mM for rooster [34], 10 mM for bull [10], 20 mM for dog [35], 20 mM for rabbit^11^  , 25 mM for buck [28], 50 mM for stallions [29], and 80 mM for man [36] and donkey [37]. Glutamine at a concentration of 18 mM was related to protecting mammalian cells against freeze-thaw damage in balanced salt solutions. The lowest concentration level of glutamine, which was a better protector than proline and betaine, was 20 mM [38]. In liquid preservation, the result revealed that supplementation of 20 mM glutamine to the BTS significantly improved (*P* < 0.05) the motility, acrosome integrity, and membrane integrity of boar sperm [18]. However, the results from this study indicated that glutamine supplementation at a higher concentration, such as the 100 mM glutamine supplementation group, in a short-term semen extender at a 17 °C holding time may cause toxicity from osmotic effects similar to the previous report by Kruuve et al. [39], Trimeche et al. [38], and Wang et al. [18].

The freezing process induces capacitated sperm as a consequence of the loss of cholesterol from the plasma membrane [4]. The capacitation status of sperm can be assessed by observing the calcium-mediated changes using fluorescent CTC [40], but the color of this reaction remains somewhat unclear. Moreover, 6-CFDA has been used to identify viable cells by permeating across intact live cell membranes. Thus, the novel combination of CTC/6-CFDA/EthD-1 in this experiment easily classified the four patterns by fluorescing brighter than the use of CTC alone. Our results demonstrate that the F pattern was significantly greater in the 20 mM glutamine supplementation group after thawing and storing for 2 h. Oh et al. [40] revealed that the farrowing rate was significantly correlated with the percentage of F-pattern spermatozoa, VCL, and LIN. In terms of the percentage of AR pattern spermatozoa, this was negatively correlated with the farrowing rate.

The holding time in a short-term semen extender of boar semen before the freezing process has a beneficial effect because it has been noted that boar sperm incubated with seminal plasma improves the resistance of sperm against cold shock and reverses the sperm capacitation process [41, 42]. The addition of 20 mM glutamine in a short-term semen extender (Bio Pig^®^) with boar semen has an advantageous effect on seminal plasma to support boar sperm membranes against ROS-induced damage by decreasing sperm cryoinjury and increasing the sperm motility and quality of the frozen-thawed boar semen.

In general, glutamine is essential for GSH production, as it is metabolized through the gamma-glutamyl cycle to generate glutathione, a key antioxidant in semen that provides intracellular protection to sperm against oxidative stress [9]. Previous studies, such as Wang et al. (2018) [18], have demonstrated that a BTS extender supplemented with glutamine offers enhanced protection to boar sperm from oxidative stress by promoting GSH synthesis during liquid storage. Similarly, Zhu et al. (2017) [11] reported that supplementing a freezing extender with 20 mM glutamine in rabbits improved glutathione content and the activity of gamma-glutamyl cysteine synthetase and glutathione peroxidase during cryopreservation and post-thaw incubation. This supplementation also reduced ROS levels in sperm throughout cryopreservation and post-thaw incubation [11]. The limitation of the present study was the absence of an investigation into the metabolic function of glutamine, specifically its role in glutathione (GSH) synthesis. Therefore, the metabolic function for understanding glutamine’s potential antioxidant effects and protective mechanisms in sperm preservation should be investigated in further study.

## Conclusion

Supplementation of glutamine at a concentration of 20 mM in a short-term boar semen extender (Bio Pig^®^) during the holding time at 17 °C before cryopreservation, which had a standard freezing extender (TEY; 9.0% glycerol and 1.9% Equex paste), provided the greatest results after thawing. It enhanced the post-thaw sperm motility and quality parameters. This research reveals a valuable improvement in boar semen cryopreservation for sperm survival from cold shock. Thus, it may improve fertility through artificial insemination with frozen-thawed boar semen and support further development in the boar industry for the storage and transfer of good genetic traits.

## Data Availability

No datasets were generated or analysed during the current study.

## Abbreviations

CASA: Computer-assisted sperm analysis system
TEY: Tris-egg yolk
